# Supplementary material for: SARS-CoV-2 B.1.617.2 Delta variant replication and immune evasion
Source: Nature. Author manuscript; Available in PMC 2021 Nov 10. (PMC8566220; doi:10.1038/s41586-021-03944-y)
Supplement: Supplementary Information [file EMS137317-supplement-Supplementary_Information.pdf]

nature portfolio

https://doi.org/10.1038/s41586-021-03944-y

Supplementary information

SARS-CoV-2 B.1.617.2 Delta variant replication and immune evasion

In the format provided by the authors and unedited
