# Supplementary material for: SARS-CoV-2 B.1.617.2 Delta variant replication and immune evasion
Source: Nature. 2021 Sep 6;599(7883):114–9. doi: 10.1038/s41586-021-03944-y (PMC8566220; doi:10.1038/s41586-021-03944-y)
Supplement: Supplementary file 1 [file 41586_2021_3944_MOESM2_ESM.pdf]

nature research

Corresponding author(s): Ravindra K Gupta (rkg20@cam.ac.uk)

Last updated by author(s): Aug 13, 2021

Reporting Summary

Nature Research wishes to improve the reproducibility of the work that we publish. This form provides structure for consistency and transparency in reporting. For further information on Nature Research policies, see our Editorial Policies and the Editorial Policy Checklist.

Statistics

For all statistical analyses, confirm that the following items are present in the figure legend, table legend, main text, or Methods section.

n/a	Confirmed	
☐	☒	The exact sample size (*n*) for each experimental group/condition, given as a discrete number and unit of measurement
☐	☒	A statement on whether measurements were taken from distinct samples or whether the same sample was measured repeatedly
☐	☒	The statistical test(s) used AND whether they are one- or two-sided<br>*Only common tests should be described solely by name; describe more complex techniques in the Methods section.*
☐	☒	A description of all covariates tested
☒	☐	A description of any assumptions or corrections, such as tests of normality and adjustment for multiple comparisons
☐	☒	A full description of the statistical parameters including central tendency (e.g. means) or other basic estimates (e.g. regression coefficient) AND variation (e.g. standard deviation) or associated estimates of uncertainty (e.g. confidence intervals)
☐	☒	For null hypothesis testing, the test statistic (e.g. *F*, *t*, *r*) with confidence intervals, effect sizes, degrees of freedom and *P* value noted<br>*Give P values as exact values whenever suitable.*
☒	☐	For Bayesian analysis, information on the choice of priors and Markov chain Monte Carlo settings
☒	☐	For hierarchical and complex designs, identification of the appropriate level for tests and full reporting of outcomes
☐	☒	Estimates of effect sizes (e.g. Cohen's *d*, Pearson's *r*), indicating how they were calculated

Our web collection on statistics for biologists contains articles on many of the points above.

Software and code

Policy information about availability of computer code

Data collection	Graphad Prism v9.0.2 were used to produce figures.<br>Mafft v7.475 was used for multiple sequence alignments.<br>IQTREE and ModelFinder v2.1.4 was used to infer maximum-likelihood phylogenies.<br>R v4.1.0 and ggplot package v3.3.3 were used to annotate phylogenies.
Data analysis	NextClade server v0.14.4 and Pangolin v3.0.5 were used to assign lineages to sequences.<br>Pymol Graphics Suite v2.4.0 was used to visualize and annotate 3D protein structures<br>Stata v13 was used for statistical analyses.

For manuscripts utilizing custom algorithms or software that are central to the research but not yet described in published literature, software must be made available to editors and reviewers. We strongly encourage code deposition in a community repository (e.g. GitHub). See the Nature Research guidelines for submitting code & software for further information.

Data

Policy information about availability of data

All manuscripts must include a data availability statement. This statement should provide the following information, where applicable:

- Accession codes, unique identifiers, or web links for publicly available datasets
- A list of figures that have associated raw data
- A description of any restrictions on data availability

Sequences from SARS-CoV-2 were obtained from GISAID database (https://gisaid.org/) using the filters and search parameters defined in the methods section.
Structural models were obtained from the Protein Data Bank (PDB) https://www.rcsb.org/.
All fasta consensus sequences files donated by collaborators are freely available from Gisaid (https://gisaid.org) with accession numbers as follows: Hospital 1:

EPI_ISIL_1970102 – EPI_ISIL_17010116; Hospital 2: EPI_ISIL_2461070 – EPI_ISIL_2955768; Hospital 3: EPI_ISL_2955782 - EPI_ISL_3066853; or from https://github.com/Steven-Kemp/hospital_india/tree/main/consensus_fasta. A list of anonymised IDs and their corresponding Gisaid accession can be found at the github link. All consensus sequence data was additionally submitted to NCBI Genbank and can be found with the following accession numbers: MZ724413 - MZ724540.

Field-specific reporting

Please select the one below that is the best fit for your research. If you are not sure, read the appropriate sections before making your selection.

☒ Life sciences ☐ Behavioural & social sciences ☐ Ecological, evolutionary & environmental sciences

For a reference copy of the document with all sections, see nature.com/documents/nr-reporting-summary-flat.pdf

Life sciences study design

All studies must disclose on these points even when the disclosure is negative.

Sample size	This is a descriptive study of an outbreak of SARS-CoV-2 in 3 hospitals and so a sample size is not appropriate.
Data exclusions	For sequencing data, consensus fasta files were excluded, as described in the methods section, based on poor coverage of the genomes.
Replication	Experiments were done in technical duplicates and each experiment was repeated.
Randomization	Not applicable as this is not an intervention study.
Blinding	Patients were pseudoanonymised at point of receiving all data from collaborators. All reported data in phylogenies are blinded and only show values and lineage assignments.

Reporting for specific materials, systems and methods

We require information from authors about some types of materials, experimental systems and methods used in many studies. Here, indicate whether each material, system or method listed is relevant to your study. If you are not sure if a list item applies to your research, read the appropriate section before selecting a response.

Materials & experimental systems

n/a	Involved in the study	
☐	☒	Antibodies
☐	☒	Eukaryotic cell lines
☒	☐	Palaeontology and archaeology
☒	☐	Animals and other organisms
☐	☒	Human research participants
☒	☐	Clinical data
☒	☐	Dual use research of concern

Methods

n/a	Involved in the study	
☒	☐	ChIP-seq
☒	☐	Flow cytometry
☒	☐	MRI-based neuroimaging

Antibodies

Antibodies used	anti-SARS-CoV-2 Spike (Invitrogen, PA1-41165, 1:5,000)<br>anti-GAPDH (Proteintech,60004, 1:5,000)<br>anti-p24 (NIBSC, ARP365 and ARP366, 1:500; ARP313, 1in10,000)<br>anti-rabbit HRP (Cell Signaling, 7074S, 1:3,000)<br>anti-b-actin HRP (Abcam, ab8226, 1:10, 000)
Validation	Validation was conducted by manufacturers prior to sale. No further validation was undertaken.

Eukaryotic cell lines

Policy information about cell lines

Cell line source(s)	HeLa ACE2 cells were donated by kind request from James Voss as noted in the methods section.<br>HEK 293T CRL-3216 cells from ATCC were used for transfection work.<br>H1299 cells were a gift from Simon Cook as noted in methods.<br>Calu-3 cells were a gift from Paul Lehner as mentioned.<br>A549 ACE2T/TMPRSS2 cells were a kind gift from Massimo Palmerini.<br>Vero E6 ACE2/TMPRSS2 cells were a gift from Emma Thomson.<br>HAE cells (MucilAir™) were purchased from Epithelix.

	293T cells were purchased from Takara Bio (# 632180)<br>Airway epithelial organoids were prepared and donated by Joo-Hyeon Lee as described in (10.1016/j.stem.2020.10.004)
Authentication	None of the cell lines used were authenticated.
Mycoplasma contamination	All cell lines used were tested (by PCR) and were mycoplasma free.
Commonly misidentified lines<br>(See ICLAC register)	No commonly misidentified lines were used in this study.

Human research participants

Policy information about studies involving human research participants

Population characteristics	Participants include health care workers involved in an outbreak of SARS-CoV-2 in 3 hospitals in India.<br>Vaccine sera were obtained from participants involved.
Recruitment	As part of routine testing, venous serum samples were collected from the participants enrolled in the NIHR BioResource Centre Cambridge
Ethics oversight	Ethical approval for use of serum samples. Controls with COVID-19 were enrolled to the NIHR BioResource Centre Cambridge under ethics review board (17/EE/0025). Convalescent sera from healthcare workers at St. Marys Hospital at least 21 days since PCR678 confirmed SARS-CoV-2 infection were collected in May 2020 as part of the REACT2 study with ethical approval from South Central Berkshire B Research Ethics Committee (REC ref: 680 20/SC/0206; IRAS 283805). Studies involving health care workers (including testing and sequencing of respiratory samples) were reviewed and approved by The Institutional Human Ethics Committees of NCDC and CSIR-IGIB(NCDC/2020/NERC/14 and CSIR-IGIB/IHEC/2020-21/01). All participants provided informed consent.

Note that full information on the approval of the study protocol must also be provided in the manuscript.
